# Cardiometabolic Morbidity and Other Prognostic Factors for Mortality in Adult Hospitalized COVID-19 Patients in North Jakarta, Indonesia

**DOI:** 10.5334/gh.1019

**Published:** 2022-02-18

**Authors:** Arvin Pramudita, Siti Rosidah, Novi Yudia, Jeffri Simatupang, Wulan Pingkan Sigit, Rita Novariani, Priscilia Myriarda, Bambang Budi Siswanto

**Affiliations:** 1 Department of Cardiology and Vascular Medicine, Faculty of Medicine Universitas Indonesia, National Cardiovascular Center Harapan Kita, Jakarta, ID; 2 Koja General Hospital, Jakarta, ID

**Keywords:** COVID-19, cardiometabolic, prognostic factors, mortality, Indonesia

## Abstract

**Background:**

Although there have been several studies investigating prognostic factors for mortality in COVID-19, there have been lack of studies in low- and middle-income countries, including Indonesia. To date, the country has the highest mortality rate among Asian countries.

**Objective:**

We sought to identify the prognostic factors of mortality in hospitalized patients with COVID-19 in Jakarta.

**Methods:**

In this retrospective cohort study, we included all adult inpatients (≥18 years old) with confirmed COVID-19 from Koja General Hospital (North Jakarta, Indonesia) who had been hospitalized between March 20^th^ and July 31^st^, 2020. Demographic, clinical, laboratory, and radiology data were extracted from the medical records and compared between survivors and non-survivors. Univariate and multivariate logistic regression analysis were used to explore the prognostic factors associated with in-hospital death.

**Results:**

Two hundred forty-three patients were included in the study, of whom 32 died. Comorbid of hypertension (OR 3.59; 95% CI 1.12–11.48; p = 0.031), obesity (OR 6.34; 95% CI 1.68–23.98; p = 0.007), immediate need of HFNC and/or IMV (OR 64.93; 95% CI 11.08–380.61; p < 0.001), abnormal RDW (OR 3.68; 95% CI 1.09–12.34; p = 0.035), ALC < 1,000/µL (OR 3.51; 95% CI 1.08–11.44; p = 0.038), D-dimer > 500 ng/mL (OR 9.36; 95% CI 1.53–57.12; p = 0.015) on admission, as well as chloroquine treatment (OR 3.61; 95% CI 1.09–11.99; p = 0.036) were associated with greater risk of overall mortality in COVID-19 patients. The likelihood of mortality increased with increasing number of prognostic factors.

**Conclusion:**

The potential prognostic factors of hypertension, obesity, immediate need of HFNC and/or IMV, abnormal RDW, ALC < 1,000/µL, D-dimer > 500 ng/mL, and chloroquine treatment could help clinicians to identify COVID-19 patients with poor prognosis at an early stage.

## Introduction

Coronavirus disease 2019 (COVID-19) caused by severe acute respiratory syndrome coronavirus 2 (SARS-CoV-2) was first reported in December 2019 in Wuhan, China. The first confirmed case of coronavirus disease 2019 (COVID-19) in Indonesia was reported on March 2, 2020. Soon after, the disease has spread rapidly within the country. By the end of 2020, approximately 735,000 cases had been diagnosed with 22,138 deaths in Indonesia [1].

Data from Chinese Center for Disease Control and Prevention (CCDC), including more than 72,000 people with COVID-19 from the country, showed that 81% were mild (absent or mild pneumonia), 14% were severe (hypoxia, dyspnea, >50% lung involvement within 24–48 hours), 5% were critical (shock, respiratory failure, multiorgan dysfunction), and 2.3% were fatal [2]. A number of factors associated with mortality have been identified from China, such as older age, male sex, presence of comorbidities, and abnormal lab findings (high WBC, high LDH, high procalcitonin, high D-dimer, low albumin level) [2,3,4,5,6].

To date, Indonesia has the highest mortality rate among Asian countries with third most confirmed cases of COVID-19 after India and Iran [1]. As part of low- and middle-income countries (LMICs), this situation represents a big challenge for Indonesia with its constrained critical care capacity to treat COVID-19 and limited financial resources [7]. Despite of that, little is known about the prognostic factors contributing to the high mortality rate in Indonesia. Understanding these factors is crucial, not only for early detection of high-risk patient in the country’s hospital setting, but also to guide local authorities developing appropriate policies to avoid the collapse of the healthcare system. Hence, this study was performed to identify the prognostic factors of mortality in hospitalized patients with COVID-19 in Jakarta, the capital city of Indonesia.

## Methods

### Study design

This retrospective cohort study was conducted in Koja General Hospital, a tertiary and one of COVID-19 referral hospital in Jakarta, Indonesia. All consecutive adult patients (age ≥ 18 years) diagnosed with COVID-19 and hospitalized between March 20^th^ and July 31^st^, 2020 were enrolled. A confirmed case of COVID-19 was defined as a positive result on real-time reverse transcriptase polymerase chain reaction (RT-PCR) for the presence of SARS-CoV-2 in nasopharyngeal swab specimens [8]. Patients who were referred to other hospital or still on treatment were excluded.

### Data collection

Demographic, clinical, laboratory, and radiology data were extracted from medical records of the participants. Demographic and clinical data included age, sex, symptoms on admission (cephalgia, fever, cough, dyspnea, dysphagia, rhinorrhea, chest pain, nausea, diarrhea, dyspepsia), comorbidities (hypertension, cardiovascular disease, diabetes mellitus, chronic kidney disease, asthma, tuberculosis, and obesity), vital signs on admission (blood pressure and heart rate), immediate need of supplemental oxygen, and treatment in the hospital (antibiotics, oseltamivir, and chloroquine). Laboratory data consisted of complete blood count, blood biochemistry, C-reactive protein (CRP) and D-dimer taken on the first day of admission. Chest X-ray was also taken on the first day and interpreted by a radiologist, seeking for cardiomegaly as the only indicator that could be measured objectively. The end-point of our study was overall in-hospital mortality rate, regardless of length of stay or cause of death.

### Statistical analysis

For the statistical analysis, age was categorized into (1) <45 years old and (2) ≥45 years old, following previous similar study in Indonesia [9]. Symptoms on admission, comorbidities, vital signs on admission, cardiomegaly, and treatment options in the hospital were recorded as (1) yes or (2) no. Obesity was defined as body mass index (BMI) ≥25 kg/m^2^ based on the guideline from World Health Organization for Asia-Pacific population [10]. Blood pressure (BP) on admission was categorized using the cut-off of 140/90 mmHg [11]. Immediate need of supplemental oxygen was based on clinical judgement of the physician to maintain patients’ normal peripheral oxygen saturation and was recorded as (1) immediate need of high flow nasal cannula (HFNC) and/or invasive mechanical ventilation (IMV), (2) immediate need of nasal cannula up to non-rebreather mask (NRM), or (3) no immediate need of oxygen. Statistically significant hematological values were categorized as (1) normal and (2) abnormal based on reference values used at our hospital. Categories for neutrophil-to-lymphocyte ratio (NLR) were (1) >3.13 and (2) ≤3.13 [12]. Categories for absolute lymphocyte count (ALC) were (1) <1,000/µL and (2) ≥1,000/µL [13]. Categories for CRP were (1) >10 mg/dL and (2) ≤10 mg/dL [14]. Categories for D-dimer were (1) >500 mg/dL and (2) ≤500 mg/dL [14].

Continuous variables are expressed as means ± standard deviation or median [range] and were compared by Student’s *t*-test or the Mann-Whitney U test. Categorical variables are described as number (%) and were compared by the χ2 test or Fisher’s exact test. Univariate logistic regression analysis was performed to identify prognostic factors of mortality. Multivariate logistic regression analysis was conducted with variables that showed p < 0.05 in univariate analysis. In all analyses, two-tailed p < 0.05 was taken to indicate statistical significance. All statistical analyses were performed using SPSS software (ver. 25.0; IBM, North Castle, NY, USA).

### Ethical statement

This study was reviewed and approved by the Ethical Committee of Koja General Hospital (04/KOMEP/2020). The requirement for informed consent was waived because of the retrospective study design. The final follow-up date was July 31^st^, 2020.

## Results

**Figure 1:**
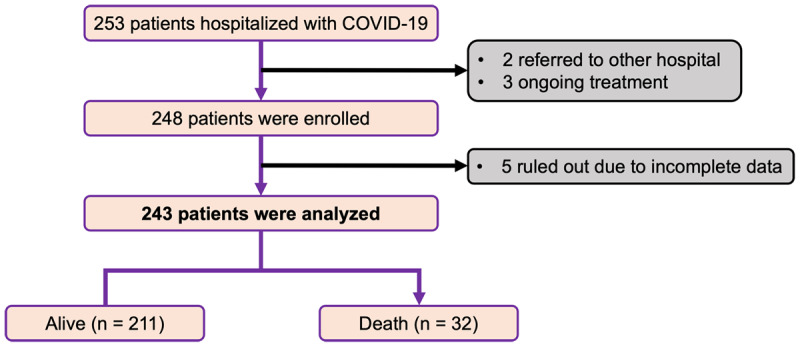
Flowchart. COVID-19 = coronavirus disease 2019.

There were 253 hospitalized patients with confirmed COVID-19 throughout our time period. Five cases were excluded as 2 patients were transferred to other hospital by patients’ preference and 3 still hospitalized as of July 31^st^, 2020. A total of 248 patients were enrolled, of whom 243 cases were included in the study. Five cases were excluded due to incomplete key information in their medical records (***Figure 1***).

Baseline characteristics of all patients are summarized in ***Table 1***. The patients in the death group were significantly older than the patients in the alive group (54.2 ± 14 vs. 47.1 ± 14.3, respectively, p = 0.009). There was no difference in sex between both groups. The death group was significantly more likely to have comorbidities of hypertension (53.1% vs. 29.4%, respectively, p = 0.008), diabetes mellitus (43.8% vs. 17.1%, respectively, p = 0.001) and obesity (37.5% vs. 14.2%, respectively, p = 0.001). On admission, symptom of dyspnea (56.3% vs. 35.5%, respectively, p = 0.025) and radiologic findings of cardiomegaly (40.6% vs. 20.4%, respectively, p = 0.011) were significantly more common in the death group than the alive group. More patients in the death group also needed immediate oxygen supplementation than those in the alive group significantly (p < 0.001). Chloroquine was given more frequently in the death group significantly (65.6% vs. 45%, respectively, p = 0.030).

**Table 1:** Baseline characteristics of the study participants with COVID-19.


CHARACTERISTICS	DEATH (N = 32)	ALIVE (N = 211)	*P* VALUE

Age			

Mean ± SD (years)	54.2 ± 14	47.1 ± 14.3	0.009

Age ≥ 45 years (%)	81.3	57.3	0.010

Sex, male (%)	53.1	53.1	0.996

Symptoms on admission			

Cephalgia (%)	15.6	23.7	0.309

Fever (%)	56.3	46	0.278

Cough (%)	71.9	55	0.072

Dyspnea (%)	56.3	35.5	0.025

Dysphagia (%)	9.4	13.7	0.496

Rhinorrhea (%)	3.1	9.5	0.233

Chest pain (%)	6.3	3.8	0.514

Nausea (%)	21.9	31.8	0.258

Diarrhea (%)	0	6.6	0.133

Dyspepsia (%)	25	16.1	0.215

Comorbidities			

Hypertension (%)	53.1	29.4	0.008

Cardiovascular disease (%)	25	12.8	0.067

Diabetes mellitus (%)	43.8	17.1	0.001

Chronic kidney disease (%)	9.4	3.8	0.164

Asthma (%)	0	2.8	0.425

Tuberculosis (%)	9.4	5.2	0.275

Obesity (%)	37.5	14.2	0.001

Vital signs on admission			

BP ≥ 140/90 mmHg (%)	34.4	40	0.544

Heart rates > 100 beats/min (%)	43.8	31.3	0.162

Immediate need of supplemental oxygen			<0.001

HFNC and/or IMV (%)	65.6	6.2	

Nasal cannula up to NRM (%)	28.1	29.4	

Not needed (%)	6.3	64.5	

Radiologic findings			

Cardiomegaly (%)	40.6	20.4	0.011

Treatment in hospital			

Antibiotics (%)	96.9	88.2	0.137

Oseltamivir (%)	93.8	88.6	0.382

Chloroquine (%)	65.6	45	0.030


COVID-19 = coronavirus disease 2019, SD = standard deviation, BP = blood pressure, HFNC = high-flow nasal cannula, IMV = invasive mechanical ventilation, NRM = non-rebreather mask.

Laboratory findings on hospital admission are summarized in ***Table 2***. In complete blood counts, white blood cells (WBC) count (9,700 [4,460 – 28,860] vs. 8,540 [2,790 – 35,450], respectively, p = 0.049 – 0.024) and neutrophil-to-lymphocyte ratio (NLR) (8.66 [1.91 – 30.50] vs. 3.37 [0.31 – 47.15], respectively, p < 0.001) were higher in the death group than the alive group. Absolute lymphocyte count (ALC) (1,008 [242 – 7,821] vs. 1,588 [16 – 6,219], respectively, p < 0.001) and platelet count (230,781 ± 92,319 vs. 275,110 ± 100,158, respectively, p = 0.019) were significantly lower in the death group. There was also a higher percentage of abnormal red cell distribution width (RDW) in the death group (43.8% vs. 22.9%, respectively, p = 0.021).

**Table 2:** Laboratory findings on admission in patients with COVID-19.


VARIABLES	DEATH (N = 32)	ALIVE (N = 211)	*P* VALUE

Hemoglobin (g/dL, median [range])	12.5 [7.6–16.9]	13.2 [5.7–18.3]	0.118–0.059

White blood cells (/µL)			

Median [range]	9700 [4460–28860]	8540 [2790–35450]	0.049–0.024

Distribution – abnormal WBC (%)	40.6	22.7	0.030

Hematocrite (%, median [range])	36.1 [20.9–49.7]	37.9 [17–52.1]	0.145–0.072

Platelet (/µL)			

Mean ± SD	230781 ± 92319	275110 ± 100158	0.019

Distribution – abnormal PLT (%)	21.9	13.3	0.153

Red cell distribution width (%)			

Median [range]	13.8 [11.7–19.6]	13.1 [11.3–38]	0.006–0.003

Distribution – abnormal RDW (%)	43.8	22.9	0.012

Neutrophil-lymphocyte ratio			

Median [range]	8.66 [1.91–30.50]	3.37 [0.31–47.15]	<0.001

Distribution – NLR > 3.13 (%)	84.4	54	0.001

Absolute lymphocyte count (/µL)			

Median [range]	1008 [242–7821]	1588 [16–6219]	<0.001

Distribution – ALC < 1,000 (%)	50	18.5	<0.001

Random blood glucose (mg/dL)			

Median [range]	142 [63–550]	114 [64–522]	<0.001

Distribution – RBG ≥ 200 (%)	28.1	12.3	0.018

Blood urea nitrogen (mg/dL, median [range])	51.8 [13–362.6]	21.4 [3.4–184]	<0.001

Creatinine (mg/dL)			

Median [range]	1.46 [0.59–16.12]	0.87 [0.36–105]	<0.001

Distribution – Cr > 1.2 (%)	62.5	22.7	<0.001

C-reactive protein (mg/dL)			

Median [range]	11.2 [0.38–31.29]	1.90 [0–32]	<0.001

Distribution – CRP > 10 (%)	62.5	26.5	<0.001

D-dimer (ng/mL)			

Median [range]	2922 [590–10000]	987 [141–15441]	<0.001

Distribution – D-dimer > 500 (%)	93.8	46.9	<0.001

	**n = 21**	**n = 148**	

Alanine transaminase (U/L, median [range])	38 [10–480]	28.5 [4–247]	0.06–0.03

Aspartate transaminase (U/L, median [range])	55 [15–1500]	26 [12–269]	<0.001

	**n = 32**	**n = 149**	

Sodium (mEq/L, median [range])	134 [119–159]	138 [109–152]	0.005–0.002

Potassium (mEq/L, median [range])	4.16 [2.8–8]	3.63 [1.62 -4.93]	<0.001

Chloride (mEq/L, median [range])	104 [88–115]	105 [90–114]	0.823–0.411


COVID-19 = coronavirus disease 2019, abnormal WBC = WBC < 4000 or > 11000 (/µL), abnormal PLT = PLT < 150000 or > 450000 (/µL), abnormal RDW = RDW > 14%.

With regard to blood chemistry, creatinine level was significantly higher in the death group than the alive group (1.46 [0.59 – 16.12] vs. 0.87 [0.36 – 105] mg/dL, respectively, p < 0.001)). Concentrations of blood urea nitrogen and random blood glucose were also significantly higher in the death group. Not all of the patients were assessed for their liver function and electrolyte status. From the available data (n = 169), concentrations of alanine transaminase and aspartate transaminase were significantly higher in the death group. On the other hand (n = 181), sodium level was significantly lower yet potassium level was significantly higher in the death group.

C-reactive protein, as an inflammation-related marker, was significantly higher in the death group than the alive group (11.2 [0.38 – 31.29] vs. 1.90 [0 – 32] mg/dL, respectively, p < 0.001). The same result was also applied for D-dimer (2,922 [590 – 10,000] vs. 987 [141 – 15,441] ng/mL, respectively, p < 0.001).

### Prognostic factors for mortality of COVID-19

**Figure 2:**
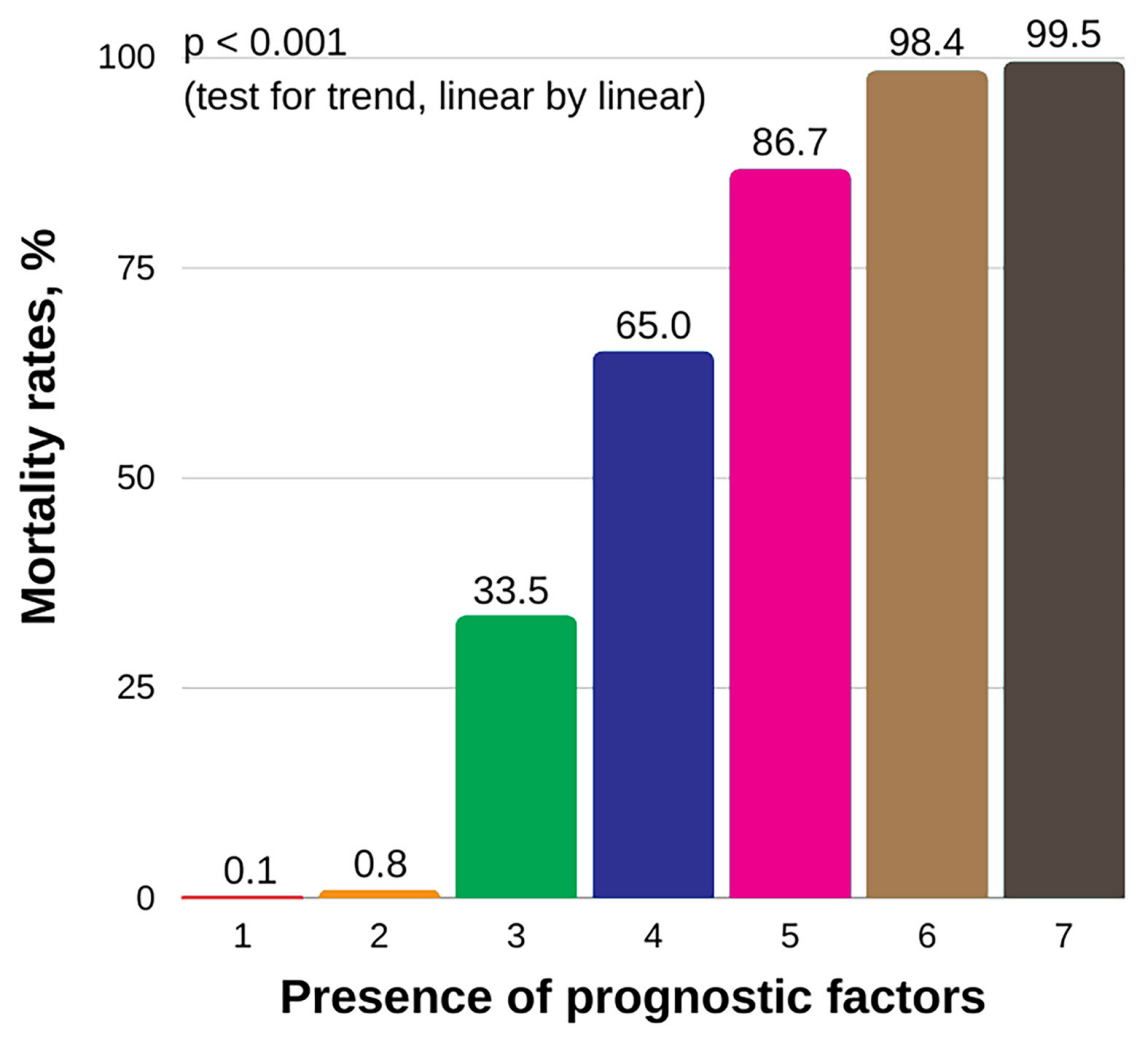
Overall mortality rates of COVID-19 according to the presence of prognostic factors. COVID-19 = coronavirus disease 2019.

Multivariate analysis with logistic regression model was performed using selected factors from univariate analysis (***Table 3***) and demonstrated that comorbid of hypertension (odds ratio [OR] 3.59; 95% confidence interval (CI) 1.12–11.48; p = 0.031), obesity (OR 6.34; 95% CI 1.68–23.98; p = 0.007), immediate need of HFNC and/or IMV (OR 64.93; 95% CI 11.08–380.61; p < 0.001), abnormal RDW (OR 3.68; 95% CI 1.09–12.34; p = 0.035), ALC < 1,000/µL (OR 3.51; 95% CI 1.08–11.44; p = 0.038), D-dimer > 500 ng/mL (OR 9.36; 95% CI 1.53–57.12; p = 0.015) on admission, as well as chloroquine treatment (OR 3.61; 95% CI 1.09–11.99; p = 0.036) were associated with greater risk of overall mortality in COVID-19 patients (***Table 4***). The likelihood of mortality increased with increasing number of prognostic factors (p < 0.001, test for trend) (***Figure 2***).

**Table 3:** Univariate analysis of prognostic factors for overall mortality in COVID-19.


PROGNOSTIC FACTORS	OR (95% CI)	*P* VALUE

Age		

≥45 years	3.22 (1.27–8.16)	0.010

<45 years	Reference	

Dyspnea		

Yes	2.33 (1.10–4.95)	0.025

No	Reference	

Hypertension		

Yes	2.72 (1.28–5.79)	0.008

No	Reference	

Diabetes mellitus		

Yes	3.78 (1.72–8.29)	0.001

No	Reference	

Obesity		

Yes	3.62 (1.60–8.16)	0.001

No	Reference	

Immediate need of supplemental oxygen		

HFNC and/or IMV	109.85 (23.13–521.70)	<0.001

Nasal cannula up to NRM	9.87 (2.07–47.04)	0.004

Not needed	Reference	

Cardiomegaly		

Yes	2.67 (1.22–5.84)	0.011

No	Reference	

Abnormal WBC		

Yes	2.32 (1.07–5.05)	0.030

No	Reference	

Abnormal RDW		

Yes	2.62 (1.22–5.66)	0.012

No	Reference	

NLR		

>3.13	4.60 (1.70–12.39)	0.001

≤3.13	Reference	

ALC		

<1,000/µL	4.41 (2.03–9.58)	<0.001

≥1,000/µL	Reference	

D-dimer		

>500 ng/mL	16.97 (3.95–72.83)	<0.001

≤500 ng/mL	Reference	

CRP		

>10 mg/dL	4.61 (2.11–10.04)	<0.001

≤10 mg/dL	Reference	

RBG		

≥200 mg/dL	2.78 (1.16–6.67)	0.018

<200 mg/dL	Reference	

Creatinine		

>1.2 mg/dL	5.66 (2.58–12.40)	<0.001

≤1.2 mg/dL	Reference	

Treatment – Chloroquine		

Yes	2.33 (1.07–5.08)	0.030

No	Reference	


COVID-19 = coronavirus disease 2019, OR = odds ratio, CI = confidence interval, HFNC = high-flow nasal cannula, IMV = invasive mechanical ventilation, NRM = non-rebreather mask, WBC = white blood cells, RDW = red cell distribution width, NLR = neutrophil-to-lymphocyte ratio, ALC = absolute lymphocyte count, CRP = C-reactive protein, RBG = random blood glucose.

**Table 4:** Multivariate logistic regression analysis of prognostic factors for mortality in COVID-19.


PROGNOSTIC FACTORS	OVERALL MORTALITY	

	OR (95% CI)	*P* VALUE

Age > 45 year	1.26 (0.30–5.28)	0.754

Dyspnea	1.58 (0.47–5.25)	0.458

Hypertension	3.59 (1.12–11.48)	0.031

Diabetes mellitus	0.69 (0.16–2.97)	0.617

Obesity	6.34 (1.68–23.98)	0.007

Oxygen – nasal cannula up to NRM	2.99 (0.54–16.42)	0.207

Oxygen – HFNC and/or IMV	64.93 (11.08–380.61)	<0.001

Cardiomegaly	1.22 (0.31–4.82)	0.781

Abnormal WBC count	1.96 (0.55–7.06)	0.301

Abnormal RDW	3.68 (1.09–12.34)	0.035

NLR > 3.13	0.48 (0.10–2.20)	0.344

ALC < 1000/µL	3.51 (1.08–11.44)	0.038

D–dimer > 500 ng/mL	9.36 (1.53–57.12)	0.015

CRP > 10 mg/dL	1.03 (0.30–3.56)	0.961

RBG ≥ 200 mg/dL	3.24 (0.76–13.92)	0.113

Creatinine > 1.2 mg/dL	1.85 (0.56–6.16)	0.316

Treatment – Chloroquine	3.61 (1.09–11.99)	0.036


COVID-19 = coronavirus disease 2019, OR = odds ratio, CI = confidence interval, HFNC = high-flow nasal cannula, IMV = invasive mechanical ventilation, NRM = non-rebreather mask, WBC = white blood cells, RDW = red cell distribution width, NLR = neutrophil-to-lymphocyte ratio, ALC = absolute lymphocyte count, CRP = C-reactive protein, RBG = random blood glucose.

## Discussion

Jakarta is the capital of Indonesia and currently has the highest mortality rate and most confirmed cases of COVID-19 in the country [15]. Moreover, it is the most populous region in Indonesia. Koja General Hospital is one of the first hospitals appointed by the local government to become a COVID-19 referral center in the area. The hospital is located in a densely populated area of Koja in the North Jakarta district which has the lowest Human Development Index among the capital’s main districts [16].

Among the 243 patients with COVID-19, the in-hospital case fatality rate was 13.7% in this study.

We showed that the presence of hypertension, obesity, immediate need of HFNC and/or IMV, abnormal RDW, ALC < 1,000/µL, D-dimer > 500 ng/mL, and chloroquine treatment were independent predictors of mortality in hospitalized adult COVID-19 patients. To our knowledge, this is one of the first studies to evaluate the prognostic factors of mortality in COVID-19 in Indonesia. It is unique in examining the combinations of demographic, clinical, laboratory, and radiological characteristics and their associations with death.

Studies from multiple countries have reported evidence that underlying cardiometabolic conditions may be associated with worse prognosis of COVID-19 [2,17,18,19,20]. Our findings supported those of previous reports from other countries, particularly hypertension and obesity, as they were associated with mortality. Diabetes was also found to be in a significantly higher proportion in our death group. However, different from other studies, it was not associated with mortality after adjustment with other prognostic factors. Cardiometabolic diseases, including hypertension, diabetes mellitus and obesity, are associated with diminished innate and adaptive immune response [21,22,23]. They are also linked with endothelial dysfunction and persistent low-grade inflammation [18]. Obesity reduces baseline pulmonary function and ventilatory reserve, which could predispose to worse COVID-19 outcomes [24]. The association between angiotensin-converting enzyme 2 (ACE2) expression and hypertension may also partly explain the high prevalence of severe COVID-19 in hypertensive patients [6]. Biologic plausibility is further supported by the unusual harms of COVID-19 related to vascular endothelial cells, in the lungs and throughout the body [25]. Overall, individuals with cardiometabolic conditions are likely predisposed to higher risks of lung injury, cytokine storm, and respiratory failure from COVID-19 infection [19,24].

These conditions also promote prothrombotic milieu as the basis for coagulopathy found in COVID-19 patients [26]. Furthermore, hypoxia-mediated hyperviscocity may also aggravate thrombosis [25,27]. Vascular injury, along with the hypercoagulability state, may aggravate the risk of cardiac injury and thus further demonstrate COVID-19 and its relationship with the heart. An increase of D-dimer level in COVID-19 patients, both at admission and during hospitalization, has been linked with increased mortality and admission to critical care [25,28]. Our study also suggested the same finding, as D-dimer > 500 ng/mL was one of the strongest predictors of mortality among other available factors.

The significant difference in immediate need of oxygen supplementation between survivors and non-survivors in our study indicates this factor is associated with the severity of illness. Interestingly, our multivariate analysis showed only the immediate need of HFNC and/or IMV as prognostic factor for mortality. Both HFNC and IMV were used in our hospital for patients with respiratory failure. Our findings confirmed reports from previous studies that the need for mechanical ventilation was associated with high mortality in COVID-19 patients [29,30,31,32]. Profound hypoxemia from respiratory failure enhances various cytotoxic functions of neutrophils and can promote hyperinflammation. Thus, it not only represents a consequence of respiratory disease but also contributes significantly to progressive lung damage after establishment of the initial injury [33,34].

Our hematological findings showed higher RDW and lymphopenia as independent predictors for mortality. RDW reflects the heterogeneity in the volume of circulating erythrocytes. In critically ill patients with sepsis, baseline RDW has been shown to be a significant and independent predictor of mortality [35]. Consistent with our findings, numerous studies have reported the association of elevated RDW with mortality in the context of COVID-19 [36,37,38]. The exact mechanism behind the association has yet to be elucidated. Multiple theoretically viable hypotheses can be made to justify the prognostic role of RDW in COVID-10, including direct cytopathic injury due to infection of circulating erythrocytes, indirect erythrocyte damage consequent to intravascular coagulopathy, dysfunctional hematopoiesis due to hyperinflammatory state, and profound disturbance of iron metabolism due to the sustained inﬂammatory response [36,39].

Lymphopenia is the most common abnormality on the complete blood count in COVID-19 patients [14,25]. Low lymphocytes are also associated with poor prognosis, with lymphocyte percentage <10% on the WBC differential is strongly associated with decreased survival [40]. A recent meta-analysis proposed that lymphopenia is an important hematological signal of severe COVID-19 and could be a practical parameter to predict severe outcomes [41]. Numerous possible explanations for lymphopenia in COVID-19 have been proposed, including destruction of lymphocytes via angiotensin-converting enzyme 2 (ACE2) receptor, lymphatic organ damage, acidemia, bone marrow suppression, and cytokine storm [28,40,42].

Chloroquine is an anti-malarial 4-aminoquinoline shown to have in vitro activity against SARS-CoV-2 and may have beneficial immunomodulatory effects in vivo [43,44]. An initial report from Gao et al. described the superiority of chloroquine for the treatment of COVID-19-associated pneumonia, compared to the control treatment, in 100 patients enrolled from 10 hospitals in China [45]. Further related study was not available yet. On the other hand, hydroxychloroquine was preferable in other studies due to its overall efficacy and safety. Still, data from previous observational studies were still inconsistent. Two large retrospective observational studies of hospitalized patients with COVID-19 reported no significant reduction in risk of in-hospital mortality for those who received hydroxychloroquine when compared to control [46,47]. Conversely, another large retrospective cohort study reported a survival benefit among hospitalized patients who received hydroxychloroquine compared to those who did not [48]. However, a substantially higher percentage of patients in the hydroxychloroquine arms also received corticosteroids (77.1% of patients in the hydroxychloroquine arms vs. 36.5% of patients in the control arm). This imbalance in corticosteroid use may confounded the findings as steroids were reported to improve the survival rate of patient with COVID-19 in the Randomised Evaluation of COVID-19 Therapy (RECOVERY) trial [49].

Our study found that the use of chloroquine was associated with in-hospital death of hospitalized COVID-19 patients. The drug was given in the form of generic chloroquine phosphate (a 250-mg tablet containing a 150-mg base equivalent) two tablets every 12 hours for 5 consecutive days. No corticosteroid was given to our patients. Several studies have reported their concerns regarding the adverse effect of this 4-aminoquinoline drug, most notably cardiovascular toxicity, i.e., QT prolongation with an increased risk of cardiac complications in an already vulnerable population [47,50]. In RECOVERY trial, the patients who received hydroxychloroquine had a longer median hospital stay and, among those who were not on invasive mechanical ventilation at the time of randomization, a higher risk of invasive mechanical ventilation or death than those who received the standard of care [51]. In another randomized controlled trial (RCT) among hospitalized patients with mild to moderate COVID-19 in Brazil, more adverse events occurred among patients who received hydroxychloroquine among those who received the standard of care [52]. An RCT of hospitalized patient with severe COVID-19 was discontinued early when preliminary results showed higher rates of mortality and QT prolongation in association with higher dose of chloroquine treatment [53].

The strength of this study is that even though this study only includes one hospital in Jakarta, Koja General Hospital is also one of the capital city referral hospital. Therefore, there are many COVID-19 patients hospitalized with different demographics that represent general population. However, in the first few months of COVID-19 pandemic in Indonesia, some patients died in the hospital with inconclusive PCR results due to the overwhelmed diagnostic facility, thus their role might be underestimated in predicting in-hospital death. A national-scale cohort study should be done to address this limitation and to obtain validation of the prognostic factors.

In conclusion, we identified seven independent predictors of mortality in hospitalized adult COVID-19 patients in Jakarta: hypertension, obesity, immediate need of HFNC and/or IMV, abnormal RDW, ALC < 1,000/µL, D-dimer > 500 ng/mL, and chloroquine treatment were independent predictors of mortality in hospitalized adult COVID-19 patients. The likelihood of mortality increased with increasing number of prognostic factors. These findings could provide valuable insight for Indonesia and other LMICs to establish effective strategies for detecting high-risk patients as early as possible and distributing healthcare resources effectively.

## Data Accessibility Statement

Datasets of clinical and laboratory data presented in the current study are available from the corresponding author on reasonable request.
